# A case of adult-onset Bartter syndrome with transient paraplegia

**DOI:** 10.1093/omcr/omaf100

**Published:** 2025-07-14

**Authors:** Shahzaib Siddiqui, Shayan Ali Irfan, Shahrukh Ahmed, Mirza Mehmood Ali Baig, Jawad Ahmed, Jawed Iqbal

**Affiliations:** Internal Medicine, Jinnah Sindh Medical University, Rafiqui H.J, Iqbal Shaheed Rd, Karachi Cantonment, Karachi 75510, Pakistan; Internal Medicine, Dow University of Health Sciences, Mission Rd, New Labour Colony Nanakwara, Karachi 74200 Pakistan; Internal Medicine, Dow University of Health Sciences, Mission Rd, New Labour Colony Nanakwara, Karachi 74200 Pakistan; Internal Medicine, Dow University of Health Sciences, Mission Rd, New Labour Colony Nanakwara, Karachi 74200 Pakistan; Internal Medicine, Northwest Health Porter, Valparaiso, Indiana, 46383, United States; Department of Communicable Disease Center, Hamad Medical Corporation, Doha, Qatar

**Keywords:** Bartter syndrome, hypokalemia, transient paraplegia, young adult, salt wasting, case-report

## Abstract

Bartter syndrome is a rare inherited renal tubular disorder characterised by metabolic alkalosis, hyperaldosteronism, and salt wasting due to defective salt reabsorption in the ascending loop of Henle. The disease is dominant in the antenatal and neonatal populations and rare in adults. Only a small number of studies present adult-onset Bartter syndrome. We present a case of an adult with transient paraplegia diagnosed with Bartter syndrome. A 22-year-old man presented with sudden bilateral lower limb weakness and cramps for two days, with no history of diarrhoea, vomiting, diuretics, laxatives, or addiction. Biochemical studies revealed hypokalemia, metabolic alkalosis, raised urinary potassium levels and raised serum renin. The potassium replacement therapy was initially ineffective in improving symptoms. The raised urinary calcium to creatinine ratio and normal serum magnesium level were noted. There was no nephrocalcinosis, unlike previously reported cases.

## Introduction

Bartter syndrome (BS) is a rare inherited renal tubular disorder, with a prevalence of 1:1000000, identified by Bartter et al. [1]. The syndrome is characterised by hypokalemia, metabolic alkalosis, hyperaldosteronism, and salt wasting caused by defective salt reabsorption in the thick ascending limb (TAL) of the loop of Henle [2]. In infants, polyuria, polydipsia, failure to thrive, normal blood pressure, muscle weakness and cramps, and growth and mental retardation are the prominent clinical features [3]. BS dominantly presents in the antenatal and neonatal population and is quite rare in adults [4]. Currently, there is no cure for BS. However, treatment with Potassium supplements and potassium-sparing diuretics improves the symptoms [2].

Only a few case reports regarding adult BS have been published. [4–13]. Literature regarding the incidence of adult BS from Pakistan is scarce [12]. Here, we present a case of an adult with idiopathic BS in Pakistan without nephrocalcinosis.

**Table 1:** Laboratory investigations.

Laboratory Investigations	Day 1	Week 1	Week 3	Normal Range
Serum urea nitrogen (mg/dL)	29	24	28	10.0–50
Serum creatinine (mg/dL)	1.07	0.79	0.7	0.5–1.5
Serum sodium (mEq/L)	137	138	136	136–149
Serum potassium (mEq/L)	1.1	2.7	2.4	3.8–5.2
Serum chloride (mEq/L)	100	101	103	98–107
Serum bicarbonate (mEq/L)		24		25–29
Serum magnesium (mg/dL)	1.66	1.71		1.6–2.6
Serum calcium (mg/dL)	8.86			8.1–10.4
Serum phosphorus (mg/dL)	1.88			2.5–4.8
Serum glucose (mm/dL)	114		85	80–180
Renin activity (uIU/mL)		62		2.8–39.9
Serum aldosterone (ng/dL)			6.5	2.0–9
Blood pH		7.452		7.35–7.45
pCO2 (mmHg)		30.5		35–45
pO2 (mmHg)		113		80–100
Urinary potassium (mEq/L)	70.61	72.41	49.07	20–67
Urinary sodium (mEq/L)	122.71		118.54	54–150
Urinary chloride (mEq/L)	121.48			46–168
Urinary Ph	6			6.0–7.5
Proteinuria	Nil			
Hematuria Urinary Calcium/creatinine Urine volume (ml/24 hrs)	Nil 0.39 3200			>0.22 800–2000

## Case presentation

A 22-year-old Pakistani man presented to the emergency department with a two-day history of progressive bilateral lower limb weakness and cramps. He denied any history of vomiting, diarrhoea, or recent illness. However, he reports having intermittent limb weakness and cramps for several months, associated with a positive history of polyuria and constipation. The drug history was noncontributory. There was no family history of a similar condition. On examination, he exhibited flaccid paralysis of both lower limbs, with motor power reduced to 2/5. The rest of the examination was unremarkable, with no hearing deficit.

The biochemical investigation revealed hypokalemia (1.1 mEq/L) and metabolic alkalosis (pH 7.45). The urinary potassium level (70.61 mEq/L) and serum renin (62 uIU/mL) concentration were raised (Table 1). The immunofluorescence test was conducted, and the ANA, AMA, and ASMA antibodies were negative. The liver function test (LFT) and thyroid function test (TFT) were noncontributory. The polyuria (3200 mL/24 hrs), normal serum magnesium (1.66 mg/dL), and raised urinary calcium to creatinine ratio (0.39 mmol/mmol). The ultrasound kidneys showed normal-sized and shaped kidneys, with regular outer margins and normal corticomedullary distinction without stones, nephrocalcinosis, or hydronephrosis.

Upon admission, the potassium replacement therapy improved the symptoms; however, the serum potassium concentration was not improved. The renal potassium wasting with polyuria, normal serum magnesium, raised urinary calcium to creatinine ratio and raised renin concentration suggested the clinical diagnosis of BS rather than Gitelman syndrome.

Due to a lack of molecular testing, the type of BS could not be established. Potassium replacement therapy, Potassium chloride (KCl) supplements, spironolactone (50 mg), and indomethacin (25 mg) were used for the disease management.

After two months of therapy, the potassium concentration was in the normal range (3.9 mEq/L), and the patient was discharged. The KCl supplements and indomethacin were prescribed on discharge, and the patient was followed every three months for one year. No complaint was reported during the following period.

## Discussion

The mutation in genes encoding for the ion channel proteins results in defective sodium chloride reabsorption in TAL; therefore, increasing aldosterone concentration, promoting potassium, and hydrogen wasting results in metabolic alkalosis and hypokalemia [14].

There are five types of BS. Type I results from a mutation in the SLC12A1 gene in the neonatal population with polyhydramnios, nephrocalcinosis, hypokalemic alkalosis, and hyposthenuria. Type II results from KCNJ1 gene mutation involving neonatal population with polyhydramnios, nephrocalcinosis, hypokalemic alkalosis, hyposthenuria, and transient hyperkalemia. Type III results from CLCKB gene mutation with hypokalemia, nephrocalcinosis, and hypochloremic alkalosis. This type is also called the classic BS (cBS). Type IVa and IVb involve mutation BSND and CLCNKA genes, respectively, with polyhydramnios, sensory deafness, hypokalemia, and hypochloremic alkalosis as prominent features. The autosomal dominant hypocalcemia hypercalciuria (type V) results from a gain-of-function mutation in the gene encoding calcium-sensing receptor (CaSR); hypocalcemia and hypomagnesemia are prevalent in this type [2, 14].

In our clinical setting, the facility for molecular testing was not available. The patient’s symptoms and biochemical profile resembled the clinical diagnosis of cBS [11]. However, the atypical symptoms, such as the absence of nephrocalcinosis and disease occurrence in late childhood, do not correlate with the typical clinical presentation of cBS. Previously published case reports show type II and type VI BS in adult patients; however, hypokalemia and sensorial deafness are prominent features in type II and type VI BS, respectively [5, 7, 8].

The polyuria, the serum magnesium concentration within limits, and the raised urinary calcium to creatinine ratio suggest BS and rule out Gitelman syndrome [3]. Pseudo-Bartter syndrome is a similar condition with identical metabolic abnormalities and is dominant in adults. This condition frequently appears secondary to diuretic or laxative abuse; however, there was no history of laxative, diuretic, or herbal medicine [6, 9]. A few case reports present the BS secondary to an autoimmune condition [6, 10]. The immunofluorescence test was conducted; however, the ANA, AMA, and ASMA antibodies were not found.

For the patient’s management, the KCl supplements were given to maintain serum potassium level and spironolactone to maintain serum aldosterone concentration. Indomethacin was also included in the treatment plan because NSAIDs have been recommended as standard treatment for BS for decreasing renal prostaglandin, which is responsible for enhancing salt wasting [2, 12]. The medical management improved the symptoms; however, the serum potassium concentration remains low, even after three weeks. After two months, the serum potassium level was normal, and KCL supplements were continued for long-term management. Every 6-month follow-up was tailored for one year. The follow-up workup assesses the clinical features, biochemical workup, NSAIDs adverse effects assessment, electrocardiography and nephrological assessment, including nephrocalcinosis and urinalysis for proteinuria. The serum potassium was 3.5 mEq/L in the first six-month follow-up, and all other investigations were normal. The patient experienced no symptoms during this duration and stated that treatment had no effect on his quality of life.

An atypical aspect of our case is the absence of nephrocalcinosis, which is typically considered a hallmark of BS, particularly in types I and III. This absence adds a unique dimension to the diagnosis and emphasises the heterogeneity of BS presentations. Given that ultrasound imaging confirmed the absence of nephrocalcinosis, it further underscores the need for careful clinical strategies in similar atypical cases that employ long-term follow-up. Few cases have been reported with a similar phenotype, and only one case has been reported from Pakistan [12]. However, in all the cases mentioned earlier, nephrocalcinosis was a prominent feature [8, 11, 12].

## Conclusion

Our case report reveals a case of Bartter syndrome in an adult patient without nephrocalcinosis. Potassium-sparing diuretics, NSAIDs, and potassium replacement therapy and supplements were the drug of choice. Further research is necessary to reveal the reason for the late-onset occurrence of the disease.

## Guideline

CARE guidelines for case reports were adopted for the draft preparation.
